# Supplementary material for: Have you heard of Rift Valley fever? Findings from a multi-country study in East and Central Africa
Source: PLoS One. 2025 Jul 2;20(7):e0327398. doi: 10.1371/journal.pone.0327398 (PMC12221053; doi:10.1371/journal.pone.0327398)
Supplement: Supplementary Table 1 — (DOCX) [file pone.0327398.s001.docx]

| **Question** | **Correct answer** | **Score** |
| --- | --- | --- |
| Have you ever heard of Rift Valley Fever? | Yes | 1 |
| Can humans get RVF? | Yes | 1 |
| If yes, how does one get RVF | 8 answers | Score of 0 if no answer ticked. Score of 1 if 1 answer ticked. Score of 2 if 2 or more answers picked |
| What are the signs and symptoms of RVF in humans | 22 answers | Score of 0 if no answer ticked. Score of 1 if 3 answers ticked. Score of 2 if 4 or more answers picked |
| Is RVF preventable in humans | Yes | 1 |
| How is RVF prevented in humans | 4 answers | Score of 0 if no answer ticked. Score of 1 if 1 answer ticked. Score of 2 if 2 or more answers picked |
| Can animals get RVF | Yes | 1 |
| If yes, what do you think transmits RVF in animals | Yes | 1 |
| Can RVF be prevented in animals | Yes | 1 |
| If Yes, what methods can be used to prevent RVF in animals | 4 answers | Score of 0 if no answer ticked. Score of 1 if 1 answer ticked. Score of 2 if 2 or more answers picked |
