# Supplementary material for: Have you heard of Rift Valley fever? Findings from a multi-country study in East and Central Africa
Source: PLoS One. 2025 Jul 2;20(7):e0327398. doi: 10.1371/journal.pone.0327398 (PMC12221053; doi:10.1371/journal.pone.0327398)
Supplement: Supplementary Table 2 — (DOCX) [file pone.0327398.s002.docx]

| **Variable** | **Overall**, N = 4,806*^1^* | **No knowledge**, n(%) = 3,821*^1^* | **Basic knowledge**, n(%) = 786*^1^* | **Advanced knowledge**, n(%) = 199*^1^* | **cOR (95 CI)** | **p-value***^2^* |
| --- | --- | --- | --- | --- | --- | --- |
| **Country** |  |  |  |  |  | <0.001* |
| DRC | 1,370 (28.5) | 1,328 (34.8) | 7 (0.9) | 35 (17.6) | **ref** |  |
| Kenya | 1,468 (30.5) | 842 (22.0) | 548 (69.7) | 78 (39.2) | 21.4(15.7,30.0) |  |
| Uganda | 1,968 (40.9) | 1,651 (43.2) | 231 (29.4) | 86 (43.2) | 6.06(4.41,8.54) |  |
| **Age group (years)** |  |  |  |  |  | <0.001* |
| 10–20 | 925 (19.2) | 810 (21.2) | 99 (12.6) | 16 (8.1) | **ref** |  |
| 21–40 | 2,494 (51.9) | 1,964 (51.4) | 401 (51.0) | 129 (64.8) | 1.93(1.56,2.41) |  |
| Above 40 | 1,387 (28.9) | 1,047 (27.4) | 286 (36.4) | 54 (27.1) | 2.27(1.81,2.86) |  |
| **Sex** |  |  |  |  |  | <0.001* |
| Female | 2,763 (57.5) | 2,315 (60.6) | 357 (45.4) | 91 (45.7) | **ref** |  |
| Male | 2,043 (42.5) | 1,506 (39.4) | 429 (54.6) | 108 (54.3) | 1.83(1.59,2.11) |  |
| **Education Level** |  |  |  |  |  | <0.001* |
| No education | 283 (5.9) | 266 (7.0) | 15 (1.9) | 2 (1.0) | **ref** |  |
| Primary incomplete | 1,122 (23.3) | 991 (25.9) | 115 (14.6) | 16 (8.0) | 2.06 (1.26,3.59) |  |
| Primary complete | 1,640 (34.1) | 1,277 (33.4) | 317 (40.3) | 46 (23.1) | 4.37 (2.73,7.51) |  |
| Secondary complete | 1,194 (24.8) | 915 (23.9) | 224 (28.5) | 55 (27.6) | 4.78 (2.96,8.24) |  |
| Tertiary complete | 525 (10.9) | 340 (8.9) | 113 (14.4) | 72 (36.2) | 9.33(5.69,16.30) |  |
| Postgraduate | 42 (0.9) | 32 (0.8) | 2 (0.3) | 8 (4.0) | 6.02(2.45,14.30) |  |
| **Occupation** |  |  |  |  |  |  |
| Healthcare workers |  |  |  |  |  | <0.001* |
| No | 4,623 (96.2) | 3,714 (97.2) | 768 (97.7) | 141 (70.9) | **ref** |  |
| Yes | 183 (3.8) | 106 (2.8) | 18 (2.3) | 58 (29.1) | 4.42 (3.22,6.06) |  |
| Other professionals | | |  |  |  | 0.003* |
| No | 4,343 (90.4) | 3,481 (91.1) | 688 (87.5) | 174 (87.4) | **ref** |  |
| Yes | 463 (9.6) | 340 (8.9) | 98 (12.5) | 25 (12.6) | 1.45 (1.17,1.80) |  |
| Unskilled workers |  |  |  |  |  | <0.001* |
| No | 4,276 (89.0) | 3,449 (90.3) | 644 (81.9) | 183 (92.0) | **ref** |  |
| Yes | 530 (11.0) | 372 (9.7) | 142 (18.1) | 16 (8.0) | 1.68 (1.37,2.04) |  |
| Animal farmer |  |  |  |  |  | <0.001* |
| No | 4,137 (86.1) | 3,363 (88.0) | 597 (76.2) | 177 (88.9) | **ref** |  |
| Yes | 669 (13.9) | 458 (12.0) | 189 (23.8) | 22 (11.1) | 1.88 (1.58,2.25) |  |
| Crop farmer |  |  |  |  |  | 0.023* |
| No | 3,172 (66.0) | 2,529 (66.2) | 497 (63.2) | 146 (73.4) | **ref** |  |
| Yes | 1,634 (34.0) | 1,292 (33.8) | 289 (36.8) | 53 (26.6) | 1.02 (0.88,1.18) |  |
| Butcher/Slaughterhouse worker | | |  |  |  | 0.2 |
| No | 4,773 (99.3) | 3,797 (99.4) | 777 (98.9) | 199 (100.0) | **ref** |  |
| Yes | 33 (0.7) | 24 (0.6) | 7 (1.1) | 0 (0.0) | 1.35 (0.60,2.76) |  |
| Undisclosed |  |  |  |  |  | 0.7 |
| No | 4,781 (99.5) | 3,799 (99.4) | 783 (99.6) | 199 (100.0) | **ref** |  |
| Yes | 25 (0.5) | 22 (0.6) | 3 (0.4) | 0 (0.0) | 0.49 (0.12,1.41) |  |
| Student |  |  |  |  |  | 0.2 |
| No | 4,187 (87.1) | 3,323 (87.0) | 696 (88.5) | 168 (84.4) | **ref** |  |
| Yes | 619 (12.9) | 498 (13.0) | 90 (11.5) | 31 (15.6) | 0.95 (0.77,1.17) |  |
| RVF Seropositivity |  |  |  |  |  | 0.044* |
| Negative | 4,553 (94.7) | 3,623 (94.8) | 749 (95.3) | 189 (91.3) | **ref** |  |
| Positive | 253 (5.3) | 198 (5.2) | 37 (4.7) | 18 (8.7) | 1.12 (0.82,1.51) |  |
| Keeping livestock |  |  |  |  |  | <0.001* |
| No | 2,253 (46.9) | 167(21,2) | 80 (40.2) | 2006 (52.5) | **ref** |  |
| Yes | 2,553 (53.1) | 619 (78.8) | 119 (59.8) | 1,815 (47.5) | 3.18 (2.72,3.72) |  |
| Have close contact with animals | | |  |  |  | <0.001* |
| No | 495 (10.3) | 451 (11.8) | 32 (4.1) | 12 (6.0) | **ref** |  |
| Yes | 4,311 (89.7) | 3,370 (88.2) | 754 (95.9) | 187 (94.0) | 2.83 (2.09,3.94) |  |
| Slaughtering sick animal(s) | | |  |  |  | 0.081 |
| No | 241 (80.6) | 150 (76.9) | 69 (88.5) | 22 (84.6) | **ref** |  |
| Yes | 58 (19.4) | 45 (23.1) | 9 (11.5) | 4 (15.4) | 0.51 (0.21,0.92) |  |
| Drink raw milk |  |  |  |  |  | 0.021* |
| No | 4,600 (95.7) | 3,644 (95.4) | 759 (96.6) | 197 (99.0) | **ref** |  |
| Yes | 206 (4.3) | 177 (4.6) | 27 (3.4) | 2 (1.0) | 0.61 (0.40,0.90) |  |
| Mosquito prevention |  |  |  |  |  | <0.001* |
| No | 2,224 (46.3) | 1,799 (47.1) | 372 (47.3) | 53 (26.6) | **ref** |  |
| Yes | 2,582 (53.7) | 2,022 (52.9) | 414 (52.7) | 146 (73.4) | 1.2 (1.05,1.39) |  |
| Presence of swamp/quarry/irrigation scheme near residence | | | |  |  | <0.001 |
| No | 2,393 (49.8) | 2,118 (55.4) | 224 (28.5) | 51 (25.6) | **ref** |  |
| Yes | 2,413 (50.2) | 1,703 (44.6) | 562 (71.5) | 148 (74.4) | 4.82 (3.97,5.90) |  |
