# Supplementary material for: Have you heard of Rift Valley fever? Findings from a multi-country study in East and Central Africa
Source: PLoS One. 2025 Jul 2;20(7):e0327398. doi: 10.1371/journal.pone.0327398 (PMC12221053; doi:10.1371/journal.pone.0327398)
Supplement: Attachment — Submitted filename: Review of RVF Paper.docx [file pone.0327398.s005.docx]

| **Abstract** |  |  |
| --- | --- | --- |
| introduction | As stated | correction |
| Line:31 | Recently, sporadic detections among humans and animals in East Africa have replaced  large-scale outbreaks. | This statement is not correct. It is known that epizootics occur and they are related to rainfall whereas sporadic outbreaks occur in between and this sustains the virus. Please table documented and verified evidence, that one has replaced the other. |
|  |  |  |
|  |  |  |
|  |  |  |

**Have you heard of Rift Valley fever? Findings from a multi-country study in East and
Central Africa**

**Review Gitao C G**
