# Supplementary material for: Have you heard of Rift Valley fever? Findings from a multi-country study in East and Central Africa
Source: PLoS One. 2025 Jul 2;20(7):e0327398. doi: 10.1371/journal.pone.0327398 (PMC12221053; doi:10.1371/journal.pone.0327398)
Supplement: Attachment — Submitted filename: Response to Reviewers.docx [file pone.0327398.s008.docx]

**RESPONSE TO REVIEWER COMMENTS**

*Manuscript title: Have you heard of Rift Valley fever? Findings from a multi-country study in East and Central Africa*

**Reviewer 1**

1. Comment 1 (Line 23): Do you mean all the authors? If only some of the authors, then such authors should be marked as per PLOS ONE’s manuscript preparation guidelines

***Response****: Thank you for this comment. We have now specified the levels of author contributions.*

2. Comment 2 (Line 30): This statement does not read proper to me. You could revised it to ‘‘Rift Valley Fever (RVF) has caused several outbreaks across Africa………..’’ which is more straightforward

***Response:*** *Thank you for the suggestion. We agree with the reviewer that the sentence could be made more direct. We have revised it to: "Rift Valley Fever (RVF) has caused several outbreaks across Africa, impacting human health and animal trade." (Line 32-34)*

3. Comment 3 (Line 37): questions regarding their knowledge on?

***Response:*** *Thank you for the comment. We have clarified the sentence to specify that participants were asked questions regarding their knowledge of RVF transmission, symptoms, prevention, and control. The revised sentence now reads: "Sociodemographic information was collected, and participants were asked questions regarding their knowledge of RVF transmission, symptoms, prevention, and control." (Lines 39-42)*

4. Comment 4 (Line 50): You could organize the data this way, so that the adjusted odds ratio remains in parenthesis

***Response:*** *We have reorganized the data presentation as recommended by including the adjusted odds ratio within the same parenthesis as the confidence interval*

5. Comment 5 (Line 59): Do not repeat words that are already part of the title as author-suggested indexing keywords. It reduces article visibility

***Response:*** *Thank you for the insight, we have reworked this to maximize the article’s visibility. We have now excluded words already found in the title.*

**Reviewer 2**

1. Comment 1 Line 31: Recently, sporadic detections among humans and animals in East Africa have replaced large scale outbreaks

Correction “This statement is not correct. It is known that epizootics occur and they are related to rainfall whereas sporadic outbreaks occur in between and this sustains the virus. Please table documented and verified evidence, that one has replaced the other.”

***Response:*** *Thank you for the correction. We acknowledge that sporadic detections have not replaced large-scale RVF outbreaks but rather occur between major epizootics, which are often associated with periods of heavy rainfall. We have revised the sentence accordingly.
The revised sentence now reads: "Recent reports indicate sporadic detections of RVF virus among humans and animals in East Africa during inter-epidemic periods. (Lines 34-35)*

**Reviewers Comments from HTML**

**Reviewer 1:**

1. The authors can improve their manuscript by the following;
   Abstract: In the conclusion of the abstract the authors should consider rewriting looking at their findings comparing the 3 different countries they looked at.

***Response:*** *We have revised the Abstract conclusion to incorporate a comparison of findings across the three countries studied. (Lines 61-67)*

2. In the introduction, the authors mention infection through mosquito bites (Line 64, page 3), they can improve this by been exact on the type of mosquito that does the transmission of the infection.

***Response:*** *We have revised the Introduction to specify that RVF virus transmission occurs via bites from infected Aedes and Culex mosquitoes. (Line 76)*

3. Material and Methods: the authors should be exact on the types of kits used the detection of RVF in the study (line 144-145, page 7).

***Response:*** *Thank you for the comment. While our original text referenced a companion paper describing the full testing protocol, we have now added the specific test kit name to the Materials and Methods section for easier reference. Full sensitivity, specificity, and quality control procedures remain available in the cited reference [19] (Line 158-164)*

4. Conclusion: the authors should rewrite the conclusion, as it appears more as a discussion of the work now, by being more exact on their findings.

***Response:*** *We have revised the Conclusion section to focus more directly on the main findings, minimizing discussion points. (Lines 398-413)*

**Editor’s Comments**

1. Please ensure that your manuscript meets PLOS ONE's style requirements, including those for file naming. The PLOS ONE style templates can be found at

[https://journals.plos.org/plosone/s/file?id=wjVg/PLOSOne_formatting_sample_main_body.pdf](https://urldefense.com/v3/__https:/journals.plos.org/plosone/s/file?id=wjVg*PLOSOne_formatting_sample_main_body.pdf__;Lw!!JmPEgBY0HMszNaDT!uh3zi-v8eTyQconj7SAihCmvLw6XzYGF6VIqxr2bNFoobLSk_Xqt_1YDo3_rPo0LvBZM8niONDIHGAZtudEnRw$) and

[https://journals.plos.org/plosone/s/file?id=ba62/PLOSOne_formatting_sample_title_authors_affiliations.pdf](https://urldefense.com/v3/__https:/track.editorialmanager.com/CL0/https:*2F*2Fjournals.plos.org*2Fplosone*2Fs*2Ffile*3Fid=ba62*2FPLOSOne_formatting_sample_title_authors_affiliations.pdf/1/010f0196707e77bc-0edfefbd-25ed-4a84-a352-9d4cecdba378-000000/HJuD_ZM13nWlhiB5BnarzTUfAyDa5ebL6RteGyaJf2Q=208__;JSUlJSUlJQ!!JmPEgBY0HMszNaDT!uh3zi-v8eTyQconj7SAihCmvLw6XzYGF6VIqxr2bNFoobLSk_Xqt_1YDo3_rPo0LvBZM8niONDIHGAZ0V3LjCA$).

*Reponse: We have taken this into consideration.*

2. Please include a complete copy of PLOS’ questionnaire on inclusivity in global research in your revised manuscript. Our policy for research in this area aims to improve transparency in the reporting of research performed outside of researchers’ own country or community. The policy applies to researchers who have travelled to a different country to conduct research, research with Indigenous populations or their lands, and research on cultural artefacts. The questionnaire can also be requested at the journal’s discretion for any other submissions, even if these conditions are not met.  Please find more information on the policy and a link to download a blank copy of the questionnaire here: [https://journals.plos.org/plosone/s/best-practices-in-research-reporting](https://urldefense.com/v3/__https:/journals.plos.org/plosone/s/best-practices-in-research-reporting__;!!JmPEgBY0HMszNaDT!uh3zi-v8eTyQconj7SAihCmvLw6XzYGF6VIqxr2bNFoobLSk_Xqt_1YDo3_rPo0LvBZM8niONDIHGAZeyQJp0w$). Please upload a completed version of your questionnaire as Supporting Information when you resubmit your manuscript.

*Response: This has been completed and included in the manuscript after the references but also attached separately*

3. Thank you for stating in your Funding Statement:

“We acknowledge the financial support provided by the U.S. National Institute of Allergy and Infectious Diseases/National Institutes of Health (NIAID/NIH), under grant number U01AI151799, through the Centre for Research in Emerging Infectious Diseases-East and Central Africa (CREID-ECA)”

Please provide an amended statement that declares *all* the funding or sources of support (whether external or internal to your organization) received during this study, as detailed online in our guide for authors at [http://journals.plos.org/plosone/s/submit-now](https://urldefense.com/v3/__http:/journals.plos.org/plosone/s/submit-now__;!!JmPEgBY0HMszNaDT!uh3zi-v8eTyQconj7SAihCmvLw6XzYGF6VIqxr2bNFoobLSk_Xqt_1YDo3_rPo0LvBZM8niONDIHGAanMcCdLg$).  Please also include the statement “There was no additional external funding received for this study.” in your updated Funding Statement.

Please include your amended Funding Statement within your cover letter. We will change the online submission form on your behalf.

*Response:*

*Amended Funding Statement:*

*We acknowledge the financial support provided by the U.S. National Institute of Allergy and Infectious Diseases/National Institutes of Health (NIAID/NIH), under grant number U01AI151799, through the Centre for Research in Emerging Infectious Diseases-East and Central Africa (CREID-ECA). There was no additional external funding received for this study.* *The funders had no role in study design, data collection and analysis, decision to publish, or preparation of the manuscript.*

4. We note that your Data Availability Statement is currently as follows: [All relevant data are within the manuscript and its Supporting Information files.]

Please confirm at this time whether or not your submission contains all raw data required to replicate the results of your study. Authors must share the “minimal data set” for their submission. PLOS defines the minimal data set to consist of the data required to replicate all study findings reported in the article, as well as related metadata and methods ([https://journals.plos.org/plosone/s/data-availability#loc-minimal-data-set-definition](https://urldefense.com/v3/__https:/journals.plos.org/plosone/s/data-availability*loc-minimal-data-set-definition__;Iw!!JmPEgBY0HMszNaDT!uh3zi-v8eTyQconj7SAihCmvLw6XzYGF6VIqxr2bNFoobLSk_Xqt_1YDo3_rPo0LvBZM8niONDIHGAbXMlxirg$)). For example, authors should submit the following data:

- The values behind the means, standard deviations and other measures reported;

- The values used to build graphs;

- The points extracted from images for analysis.

Authors do not need to submit their entire data set if only a portion of the data was used in the reported study.

If your submission does not contain these data, please either upload them as Supporting Information files or deposit them to a stable, public repository and provide us with the relevant URLs, DOIs, or accession numbers. For a list of recommended repositories, please see [https://journals.plos.org/plosone/s/recommended-repositories](https://urldefense.com/v3/__https:/journals.plos.org/plosone/s/recommended-repositories__;!!JmPEgBY0HMszNaDT!uh3zi-v8eTyQconj7SAihCmvLw6XzYGF6VIqxr2bNFoobLSk_Xqt_1YDo3_rPo0LvBZM8niONDIHGAbV0URHsg$).

If there are ethical or legal restrictions on sharing a de-identified data set, please explain them in detail (e.g., data contain potentially sensitive information, data are owned by a third-party organization, etc.) and who has imposed them (e.g., an ethics committee). Please also provide contact information for a data access committee, ethics committee, or other institutional body to which data requests may be sent. If data are owned by a third party, please indicate how others may request data access.

*Response: All relevant data underlying the findings of this study are provided in the Supporting Information files. These include de-identified survey responses, laboratory test results, and statistical outputs necessary to replicate the reported analyses. Due to ethical restrictions regarding participant confidentiality, individual-level survey data have been de-identified. Further inquiries may be directed to the PI. After publication, the data will be further uploaded to Havard repository under WSU-GHK account.* [*https://dataverse.harvard.edu/dataverse/wsughk*](https://dataverse.harvard.edu/dataverse/wsughk)

5. Thank you for stating the following in the Acknowledgments Section of your manuscript: “We acknowledge the financial support provided by the U.S. National Institute of Allergy and Infectious Diseases/National Institutes of Health (NIAID/NIH), under grant number U01AI151799, through the Centre for Research in Emerging Infectious Diseases-East and Central Africa (CREID-ECA).”

We note that you have provided additional information within the Acknowledgements Section that is not currently declared in your Funding Statement. Please note that funding information should not appear in the Acknowledgments section or other areas of your manuscript. We will only publish funding information present in the Funding Statement section of the online submission form.

Please remove any funding-related text from the manuscript and let us know how you would like to update your Funding Statement. Currently, your Funding Statement reads as follows:

“We acknowledge the financial support provided by the U.S. National Institute of Allergy and Infectious Diseases/National Institutes of Health (NIAID/NIH), under grant number U01AI151799, through the Centre for Research in Emerging Infectious Diseases-East and Central Africa (CREID-ECA)”

Please include your amended statements within your cover letter; we will change the online submission form on your behalf.

*Response: Moved it to its own section as funding statement and also included the statement within the cover letter*

6. We note that Figure 1 in your submission contain [map/satellite] images which may be copyrighted. All PLOS content is published under the Creative Commons Attribution License (CC BY 4.0), which means that the manuscript, images, and Supporting Information files will be freely available online, and any third party is permitted to access, download, copy, distribute, and use these materials in any way, even commercially, with proper attribution. For these reasons, we cannot publish previously copyrighted maps or satellite images created using proprietary data, such as Google software (Google Maps, Street View, and Earth). For more information, see our copyright guidelines: [http://journals.plos.org/plosone/s/licenses-and-copyright](https://urldefense.com/v3/__http:/journals.plos.org/plosone/s/licenses-and-copyright__;!!JmPEgBY0HMszNaDT!uh3zi-v8eTyQconj7SAihCmvLw6XzYGF6VIqxr2bNFoobLSk_Xqt_1YDo3_rPo0LvBZM8niONDIHGAZBNwXl8A$).We require you to either (1) present written permission from the copyright holder to publish these figures specifically under the CC BY 4.0 license, or (2) remove the figures from your submission:
a. You may seek permission from the original copyright holder of Figure 1 to publish the content specifically under the CC BY 4.0 license.

We recommend that you contact the original copyright holder with the Content Permission Form ([http://journals.plos.org/plosone/s/file?id=7c09/content-permission-form.pdf](https://urldefense.com/v3/__http:/journals.plos.org/plosone/s/file?id=7c09*content-permission-form.pdf__;Lw!!JmPEgBY0HMszNaDT!uh3zi-v8eTyQconj7SAihCmvLw6XzYGF6VIqxr2bNFoobLSk_Xqt_1YDo3_rPo0LvBZM8niONDIHGAb2wV3FIg$)) and the following text: “I request permission for the open-access journal PLOS ONE to publish XXX under the Creative Commons Attribution License (CCAL) CC BY 4.0 ([http://creativecommons.org/licenses/by/4.0/](https://urldefense.com/v3/__http:/creativecommons.org/licenses/by/4.0/__;!!JmPEgBY0HMszNaDT!uh3zi-v8eTyQconj7SAihCmvLw6XzYGF6VIqxr2bNFoobLSk_Xqt_1YDo3_rPo0LvBZM8niONDIHGAbL4EwFwA$)). Please be aware that this license allows unrestricted use and distribution, even commercially, by third parties. Please reply and provide explicit written permission to publish XXX under a CC BY license and complete the attached form.”

Please upload the completed Content Permission Form or other proof of granted permissions as an "Other" file with your submission.

In the figure caption of the copyrighted figure, please include the following text: “Reprinted from [ref] under a CC BY license, with permission from [name of publisher], original copyright [original copyright year].”

b. If you are unable to obtain permission from the original copyright holder to publish these figures under the CC BY 4.0 license or if the copyright holder’s requirements are incompatible with the CC BY 4.0 license, please either i) remove the figure or ii) supply a replacement figure that complies with the CC BY 4.0 license. Please check copyright information on all replacement figures and update the figure caption with source information. If applicable, please specify in the figure caption text when a figure is similar but not identical to the original image and is therefore for illustrative purposes only.

The following resources for replacing copyrighted map figures may be helpful:

USGS National Map Viewer (public domain): [http://viewer.nationalmap.gov/viewer/](https://urldefense.com/v3/__http:/viewer.nationalmap.gov/viewer/__;!!JmPEgBY0HMszNaDT!uh3zi-v8eTyQconj7SAihCmvLw6XzYGF6VIqxr2bNFoobLSk_Xqt_1YDo3_rPo0LvBZM8niONDIHGAbcPzrCTQ$)

The Gateway to Astronaut Photography of Earth (public domain): [http://eol.jsc.nasa.gov/sseop/clickmap/](https://urldefense.com/v3/__http:/eol.jsc.nasa.gov/sseop/clickmap/__;!!JmPEgBY0HMszNaDT!uh3zi-v8eTyQconj7SAihCmvLw6XzYGF6VIqxr2bNFoobLSk_Xqt_1YDo3_rPo0LvBZM8niONDIHGAbnzXpzZg$)

Maps at the CIA (public domain): [https://www.cia.gov/library/publications/the-world-factbook/index.html](https://urldefense.com/v3/__https:/www.cia.gov/library/publications/the-world-factbook/index.html__;!!JmPEgBY0HMszNaDT!uh3zi-v8eTyQconj7SAihCmvLw6XzYGF6VIqxr2bNFoobLSk_Xqt_1YDo3_rPo0LvBZM8niONDIHGAZ8Bx26Yw$) and [https://www.cia.gov/library/publications/cia-maps-publications/index.html](https://urldefense.com/v3/__https:/www.cia.gov/library/publications/cia-maps-publications/index.html__;!!JmPEgBY0HMszNaDT!uh3zi-v8eTyQconj7SAihCmvLw6XzYGF6VIqxr2bNFoobLSk_Xqt_1YDo3_rPo0LvBZM8niONDIHGAZ5qm0s4Q$)

NASA Earth Observatory (public domain): [http://earthobservatory.nasa.gov/](https://urldefense.com/v3/__http:/earthobservatory.nasa.gov/__;!!JmPEgBY0HMszNaDT!uh3zi-v8eTyQconj7SAihCmvLw6XzYGF6VIqxr2bNFoobLSk_Xqt_1YDo3_rPo0LvBZM8niONDIHGAaSSit6ZA$)

Landsat: [http://landsat.visibleearth.nasa.gov/](https://urldefense.com/v3/__http:/landsat.visibleearth.nasa.gov/__;!!JmPEgBY0HMszNaDT!uh3zi-v8eTyQconj7SAihCmvLw6XzYGF6VIqxr2bNFoobLSk_Xqt_1YDo3_rPo0LvBZM8niONDIHGAaKeZe9hQ$)

USGS EROS (Earth Resources Observatory and Science (EROS) Center) (public domain): [http://eros.usgs.gov/#](https://urldefense.com/v3/__http:/eros.usgs.gov/*__;Iw!!JmPEgBY0HMszNaDT!uh3zi-v8eTyQconj7SAihCmvLw6XzYGF6VIqxr2bNFoobLSk_Xqt_1YDo3_rPo0LvBZM8niONDIHGAbckChEIQ$)

Natural Earth (public domain): [http://www.naturalearthdata.com/](https://urldefense.com/v3/__http:/track.editorialmanager.com/CL0/http:*2F*2Fwww.naturalearthdata.com*2F/1/010f0196707e77bc-0edfefbd-25ed-4a84-a352-9d4cecdba378-000000/Q238B5D9mUZj0DwXrynYDOaVjG6pBlaxM4AYYqTYb_8=208__;JSUl!!JmPEgBY0HMszNaDT!uh3zi-v8eTyQconj7SAihCmvLw6XzYGF6VIqxr2bNFoobLSk_Xqt_1YDo3_rPo0LvBZM8niONDIHGAauuBpCSg$)

*Response: Thank you for your attention to copyright compliance. We confirm that Figure 1 was self-generated using QGIS software, based on publicly available shapefiles from Natural Earth and GADM databases. No copyrighted basemaps such as Google Maps, Google Earth, or ESRI layers were used. The figure is therefore fully compliant with the CC BY 4.0 license.*

7. Please ensure that you refer to Figure 1 in your text as, if accepted, production will need this reference to link the reader to the figure

*Response: We have added the statement “The geographic distribution of the study areas is shown in Figure 1” under study areas to reference the figure.*

8. Please review your reference list to ensure that it is complete and correct. If you have cited papers that have been retracted, please include the rationale for doing so in the manuscript text, or remove these references and replace them with relevant current references. Any changes to the reference list should be mentioned in the rebuttal letter that accompanies your revised manuscript. If you need to cite a retracted article, indicate the article’s retracted status in the References list and also include a citation and full reference for the retraction notice.

*Response: The only change in the reference list is change of reference 19 which was initially a preprint but now a peer reviewed publication.*

Additional Comment

1. Please ensure that you refer to Table 4 in your text as, if accepted, production will need this reference to link the reader to the Table.

*Response: Thank you for catching that, I have first renamed it to table 3 then referenced it*

2. Thank you for updating your data availability statement. You note that your data are available within the Supporting Information files, but no such files have been included with your submission. At this time we ask that you please upload your minimal data set as a Supporting Information file, or to a public repository such as Figshare or Dryad. Please also ensure that when you upload your file you include separate captions for your supplementary files at the end of your manuscript.

*Response: Thank you for the request, I have uploaded the excel file in the submission system and added a caption to my manuscript.*
